# Intake Procedures in Colorado Animal Shelters

**DOI:** 10.3390/ani7050038

**Published:** 2017-05-05

**Authors:** Anna Fagre, Francisco Olea-Popelka, Rebecca Ruch-Gallie

**Affiliations:** College of Veterinary Medicine and Biomedical Sciences, Colorado State University, 300 West Drake Road, Fort Collins, CO 80523, USA; anna.fagre@colostate.edu (A.F.); francisco.olea-popelka@colostate.edu (F.O.-P.)

**Keywords:** animal shelters, infectious disease, intake procedures, *Dirofilaria immitis*, gastrointestinal endoparasites, natural disasters, transfer

## Abstract

**Simple Summary:**

Many animal shelters receive animals from different regions of the United States; particularly following natural disasters or other mass displacements. After Hurricane Katrina; Colorado experienced a surge of animal intakes from regions of the country possessing higher parasitic burden. Little is known about the extent to which shelters tailor their infectious disease screening procedures and other intake protocols based on animal origin. Using a questionnaire; shelter personnel from across the state of Colorado provided information on shelter characteristics including where they receive animals from; general intake procedures; and perceptions on infectious disease risk. We found that more shelters that take dogs in from out of state screen for heartworm and other parasitic diseases than do shelters that only take animals from within the state. No difference was seen for feline screening procedures. We also found that few shelters use widespread screening protocols and instead screen based on suspicion. Lastly; many vaccination protocols in shelters do not comply with The Association of Shelter Veterinarians Guidelines for Standards of Care in Animal Shelters. This study highlights the importance of tailoring animal intake procedures based on where the animal was transferred from.

**Abstract:**

The purpose of this study was to describe intake procedures in Colorado animal shelters, compare infectious disease screening protocols in shelters taking in animals from out-of-state to shelters only accepting animals from Colorado, and analyze perceived risk of diseases in Colorado by responding shelter personnel. A questionnaire was designed and administered to shelter personnel across the state of Colorado via the survey tool SurveyMonkey© (http://www.surveymonkey.com) or a mailed hard copy. Information collected concerned general shelter characteristics and intake procedures performed in various circumstances as reported by responding shelter personnel. Only 12.5% (5/40) of respondents reported providing core vaccines to all animals upon intake at their shelter, with young age (65.0%; 26/40), pregnancy (55.0%; 22/40), and mild existing illness (40.0%; 16/40) being cited as the top reasons for not administering core vaccines. A significantly larger proportion of shelters taking animals in from around the U.S. screened for *Dirofilaria immitis* than shelters taking in animals only from within the state of Colorado (*p* = 0.001), though a majority of respondents considered cats and dogs to be at risk of heartworm and endoparasitic infection in the state of Colorado. Based on the results of this questionnaire, relatively few shelters test dogs and cats for infectious diseases and some of those utilize tests for diagnostic purposes rather than routine screening. Additionally, vaccination protocols in several shelters are not consistent with The Association of Shelter Veterinarians Guidelines for Standards of Care in Animal Shelters. This study provides important information on intake procedures in Colorado animal shelters and highlights the importance of educating shelter staff on varying risk of infection based on the history and origin of the animal being taken in.

## 1. Introduction

Animal shelters in the state of Colorado receive a high volume of animals transferred in from around the United States, many of which are considered to pose a higher risk of infection by parasites and infectious agents not considered to be high risk within the state, such as heartworm, roundworm, hookworm, whipworm, Lyme disease, ehrlichiosis, feline leukemia, and feline immunodeficiency virus [1]. Upon intake and adoption, not only could these agents be transmitted to other animals in the community, but several possess zoonotic potential. According to the Colorado Department of Public Health and Environment, the state of Colorado saw 4 cases of human plague, 52 cases of human tularemia, and 119 cases of rabies in domestic animals and wildlife in 2015. Furthermore, a large number of animal relocation programs are in place at animal shelters in the state. An increase in out-of-state transfers is appreciated particularly during mass displacements caused by natural disasters. This is demonstrated by the increase in intakes from the gulf coast following Hurricane Katrina in 2005, a region where the average number of heartworm cases reported per clinic in some areas surpassed 100; conversely, the average number of cases reported per clinic in the state of Colorado during this time ranged from 0–5 [2].

While Colorado has not historically been considered endemic for *Dirofilaria immitis,* the causative agent of heartworm disease—changing climatic conditions—has altered competent vector population abundance and feeding behaviors, potentially contributing to future increased local transmission of *D. immitis* [3,4]. Anecdotally, several animals transported from out-of-state into Colorado have been adopted out and later discovered to be positive for heartworm. In light of these instances, it is not unreasonable to speculate that some shelters are altering intake procedures based on animal origin. As evidenced by Colby and colleagues, agencies in heartworm endemic areas lack uniformity in protocols surrounding identification, treatment, and prevention of heartworm disease due to insufficient resources [5]. Additionally, in the heartworm endemic state of Florida, sheltered dogs had a higher prevalence of *D. immitis* infection than the dog population as a whole [6]. Also of importance, the organism may affect cats in endemic areas but is rarely managed by diagnostic testing or preventive medicine due to low perceived morbidity rates, though in an 11-state survey conducted during 2005, 289 dogs and 19 cats were diagnosed with heartworm in the state of Colorado [7,8].

Following Hurricane Katrina, Levy and colleagues reported that the majority of dogs and cats rescued and transferred from the Gulf Coast disaster area demonstrated evidence of infection or exposure to at least one pathogen, many of which were zoonotic. Infectious disease screening is a critical part of infection control in any animal shelter for the community health of both humans and animals [9]. In 2011, a study by Steneroden et al. surveyed shelters across a six-state region in the Western U.S., with 45% stating that many to most animals arrive already positive for at least one infectious agent [10]. Another study indicated that almost 77% of shelter managers indicated that infectious diseases negatively impact the success of the shelter and 86.4% agreed such diseases cause financial detriment [11].

To the authors’ knowledge, the only published data on infectious disease screening procedures in animal shelters describe screening for canine heartworm, feline leukemia and/or feline immunodeficiency virus, and ringworm [12]. By examining in more detail which infectious agents are tested for upon intake and the wide range of locations animals are accepted from, a greater understanding of potential prevalence and transmission for various diseases may be gathered. This information may help shelter directors determine whether risk factor analysis for various agents based on whether the animal was received locally or from outside of the community would be an appropriate addition to intake protocols. This descriptive study highlights the importance of tailoring intake procedures based on the history and origin of the animal being transferred as shelter transfers become more commonplace in certain regions of the country. Colorado is an ideal model in which to study intake procedures, as the state receives many out-of-state intakes due to high adoption rates and a well-developed networking system between shelters in addition to a relatively low historical endemicity for many parasitic diseases due to the altitude and dry climate. The objectives of this study were to describe intake procedures in Colorado animal shelters, compare infectious disease screening protocols in shelters taking in animals from out-of-state to shelters only accepting animals from Colorado, and to analyze perceived risk of diseases in Colorado by responding shelter personnel.

## 2. Materials and Methods

A questionnaire was designed and pilot tested on a small group of shelter administrators and veterinarians (*n* = 10) prior to statewide distribution. Based on feedback, wording was changed on four questions before distribution. Shelters registered with the Pet Care Facilities Act (PACFA) Program through the Colorado Department of Agriculture were contacted via mail, email, and telephone to explain the study and ask for participation. The survey (the Supplementary Materials) was made available to 256 shelter personnel across the state of Colorado via SurveyMonkey© (http://www.surveymonkey.com) in English. Shelter personnel unavailable via phone or e-mail and those without internet access were mailed a hard copy of the survey. In total, the online/paper questionnaire required approximately 20 min of the participants’ time and approximately 30 min via phone. Reminder e-mails were sent at two and four weeks after the survey was initially provided.

Information collected included shelter demographic information, animal intake procedures (exams, vaccination, de-worming, testing, hold periods, housing, etc.), and perceived disease transmission risk analysis by shelter personnel based on whether the animal was community-received or an out-of-state transfer. Efforts were made to gain the most accurate information possible, asking respondents which geographic regions they had received animals from in the past year, whether they altered their disease screening procedures based on area of origin, and whether they consider various agents a concern in the state of Colorado. Subsequently, a descriptive analysis was performed for overall intake procedures and reported using median and 95% confidence interval of the median for continuous data. Proportions for risk perception and intake practices between shelters only taking in animals from within Colorado compared to those taking animals in nationwide were compared using the Fisher’s exact test. The level of statistical significance was <0.05. All calculations were performed using R Statistical Software© (R: The R Project for Statistical Computing, The R Foundation: Vienna, Austria). Respondents were able to skip questions if desired and thus did not need to complete the survey in its entirety prior to submission, causing the *n* to vary between questions.

## 3. Results

### 3.1. Characteristics of Responding Shelters

Of the 256 shelters contacted, respondents from 24 shelters indicated that they did not complete the questionnaire as it did not apply to their organization, leaving 232 eligible shelters. Reasons cited by respondents for the questionnaire not applying to the shelter they are employed by included the shelter’s status as a network of foster homes rather than a “brick and mortar facility” or the shelter managing species other than dogs and cats (e.g., rabbits, guinea pigs, birds, etc.). Hard copies were mailed to 16 shelters and 3 were returned. Ultimately, a response rate of 29.7% (69/232) was obtained. Of respondents, 59.3% (35/59) identified their shelter as closed admission, 28.8% (17/59) as open admission, and 11.9% (7/59) selected that they were unsure whether their shelters were open or closed admission. When asked which locations dogs and cats are accepted from, 74.2% (49/66) of respondents reported their shelters accepting animals from the municipality or county of shelter location, 53.0% (35/66) from within the entire state of Colorado, while 34.9% (23/66) of shelters stated they accept animals from across the United States and 3.0% (2/66) of respondents’ shelters accept animals from outside of the United States. Respondents were able to choose more than one option.

Of the responding shelters, a median of 355 (95% CI 250–500, range 0–36,500) animals are seen per shelter per year. Of these shelters, 67.9% (38/56) were smaller shelters (<1000 annual intakes), 25% (14/56) reported between 1000–10,000 annual intakes, and 7.1% (4/56) were moderately large shelters (>10,000 annual intakes). Of the responding shelters, the adoption or rehoming rate of healthy adoptable animals was 99% (95% CI 98.03–100%). The results of this study estimated the euthanasia rate in reporting Colorado shelters to be 1% (95% CI 0.11–4.49%). When respondents were provided a list of reasons to rank for euthanasia of intakes, the most common reason listed was aggression, followed by disease with a poor prognosis, behavioral problems other than aggression, and disease with a decent prognosis but expensive treatment. The least common reason for euthanasia was limited space and resources. However, this question did not apply to many respondents as they identified as “no-kill shelters”.

Of respondents who provided information on total number of animals examined upon intake annually at their shelter, a median of 150 (95% CI 43–279) animals were seen by a veterinarian and 40 (95% CI 1–250) were seen by a veterinary technician. In a series of questions related to staffing, 44.0% (22/50) of respondents do not have any veterinarian on staff or contract at their shelter, 22.0% (11/50) have at least one veterinarian full time, and 30.0% (15/50) have one on staff or contract part time. When asked about veterinary technicians, 65.3% (32/49) of respondents reported having none on staff or contract at their shelter, 22.4% (11/49) have at least one full-time, and 20.4% (10/49) have at least one part-time.

### 3.2. Intake Procedures

**Figure 1:**
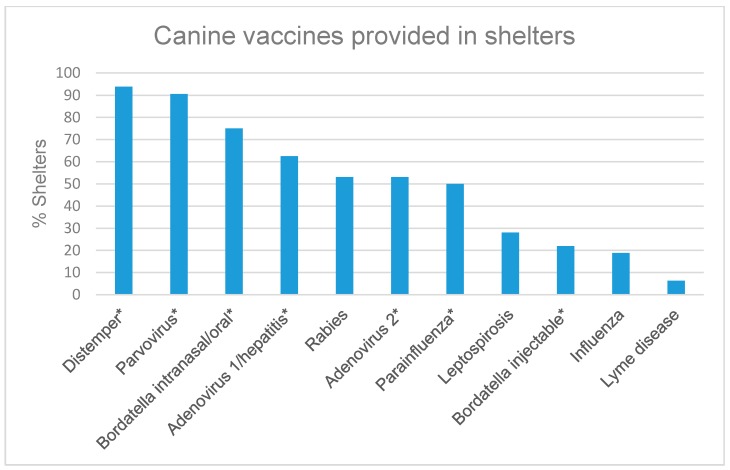
Respondents were asked which diseases they vaccinate dogs for upon intake at their shelter (*n* = 32). An asterisk (*) was used to denote diseases immunized using either a monovalent or multivalent vaccine.

**Figure 2:**
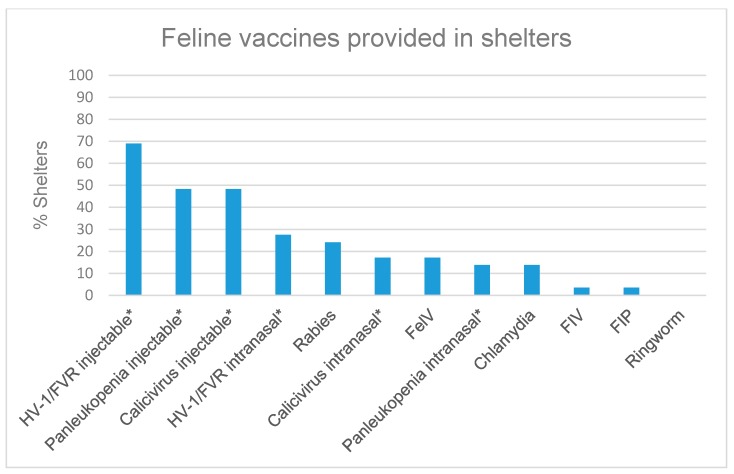
Respondents were asked which diseases they vaccinate cats against upon intake at their shelter (*n* = 29). An asterisk (*) was used to denote diseases immunized using either a monovalent or multivalent vaccine.

When provided a list of procedures to choose from that were performed immediately upon intake, 87.2% (41/47) of respondents’ shelters always attempt to locate the owner (e.g., careful screening for microchip ID), 74.5% (35/47) vaccinate, 48.9% (23/44) immediately deworm, 48.9% (23/47) have a physical examination/health evaluation performed by a non-veterinarian with documentation in record, and 38.3% (18/47) have a physical examination/health evaluation performed by a veterinarian with documentation in record. Respondents were able to choose more than one option. The most commonly cited reasons for not providing core vaccinations were that the animal was too young (65.0% (26/40)), pregnant (55.0% (22/40)), experiencing mild existing illness (40.0% (16/40)), or too old (25.0%, (10/40)). Other reasons reported were lack of funding, the animal was aggressive or fractious, the animal was stressed, the animals were vaccinated at time of adoption, or that a government contract did not require vaccination. Regardless of condition, 12.5% (5/40) of respondents reported their shelters always giving core vaccines upon intake. Of the shelters that vaccinate for rabies, 2 out of 21 indicated that they provide the vaccine upon adoption prior to the animal leaving the shelter. There was no significant difference in rabies vaccination rates between shelters with at least one veterinarian on staff or contract and those without (*p* = 0.714). The proportion of respondents vaccinating for various diseases in dogs and cats are represented in Figure 1 and Figure 2.

Not all intake procedures were performed on all animals. Respondents were asked which factors influenced shelter personnel not to perform the normal intake protocol. A list was provided and respondents were able to choose more than one option. The most commonly cited reason was the medical assessment (59.1% (13/22)), with source of animal (owner relinquished, transferred from another shelter, etc.) (40.9%, (9/22)), age of animal (31.8% (7/22)), behavioral assessment (31.8% (7/22)), reproductive status (neuter or intact) of animal (27.3% (6/22)), and animal origin (local community, out of state, etc.) (27.3% (6/22)) also being significant factors. Additionally, 5 of the 69 (7.2%) responding shelters commented that they only screen for giardia, hookworm, roundworm, whipworm, tapeworm, coccidiosis, flukes, trichomoniasis, and cryptosporidiosis in the presence of clinical signs or other suspicion of infection.

The proportion of responding shelters that screen for various diseases upon intake was stratified and compared on the basis of whether or not they accept dogs from out of state (Table 1). Respondents were also asked which diagnostic methods they use for screening (Table 2 and Table 3). Additionally, information was provided surrounding actions taken and treatment protocols upon a positive diagnosis (Table 4). Risk perception surrounding heartworm and endoparasitic disease was also compared for shelters that take animals in from out of state and those that do not (Table 5).

## 4. Discussion

Our results suggest that shelters in our study population routinely screen dogs and cats for infectious diseases upon intake and some of them utilize testing for confirmatory diagnostic purposes rather than screening purposes. Additionally, there is a significant difference in screening for heartworm disease and several gastrointestinal endoparasitic diseases (giardia, hookworm, roundworm, whipworm, and tapeworm) in dogs, but not cats, between shelters that take in animals from outside of Colorado and those that do not. Further, vaccination protocols for many respondents’ shelters were not consistent with The Association of Shelter Veterinarians Guidelines for Standards of Care in Animal Shelters.

The majority (67.9%, 38/56) of responding shelters were smaller (<1000 annual intakes) though 7.1% (4/56) took in more than 10,000 animals annually. Colorado also had a high adoption rate (99%, 95% CI 98.03–100%) and low euthanasia rate at 1% (95% CI 0.11–4.49%). These data could be in part due to statewide funded programs, including the Colorado Pet Overpopulation Fund (coloradopetfund.org) and the Pet Animal Care Facilities Act under the Colorado Department of Agriculture. It is difficult to compare these rates to a nationwide baseline due to lack of systematic data collection, though data recently released by the American Society for the Prevention of Cruelty to Animals indicate that the number of dogs and cats euthanized annually in U.S. shelters has decreased to 1.5 million in 2017 from 2.6 million in 2011, likely due to increased adoption rates and more successful programs devoted to returning stray animals to their owners [12].

Half of surveyed shelters (22/44) screen for at least one infectious disease upon intake. However, 11.4% (5/44) stated that they only use diagnostics in the face of clinical signs for at least one of the following diseases: giardia, hookworm, roundworm, whipworm, tapeworm, coccidiosis, flukes, trichomoniasis, and cryptosporidiosis. Our results suggest that shelters may make gastrointestinal endoparasitic screening decisions based on which locations they are taking dogs in from, though no significant difference was seen for feline screening protocols based on whether or not the shelter accepted out-of-state intakes (Table 1). Shelters that did not report routinely screening for endoparasites may empirically treat with a broad spectrum dewormer in lieu of performing diagnostics. A variety of anthelmentics and anti-protozoals were used in the treatment of various diseases with the most common being pyrantel and metronidazole for both dogs and cats.

Heartworm disease is perceived as a threat to the canine population of Colorado by 66.7% (26/39) respondents, with 72.7% (16/22) of responding shelters screening for heartworm disease on intake compared to another study indicating 53.9% (289/536) of shelters nationwide do so [11]. Our results demonstrate a significant difference in heartworm disease screening practices between shelters accepting dogs from outside of the state of Colorado and those only accepting dogs from within the state of Colorado, though no significant difference is seen in cat screening protocols (Table 1). These findings may indicate that shelters are making heartworm screening decisions based on which locations they are taking dogs in from. However, there was no significant difference in risk perception between respondents from shelters accepting dogs from outside of Colorado compared to respondents from shelters accepting animals only from within the state (Table 5). In shelters that do screen for heartworm disease, treatment protocols must be in place should a positive result occur. Our results indicate that this is true of our respondents, as 100% of shelters that screen dogs (16/16) and cats (2/2) have treatment protocols in place should an animal test positive.

Within the questionnaire, the definitions of “disease screening” and “disease diagnostics” were not clearly defined and may have impacted respondents’ understanding of the difference between them. It is important to note that the positive predictive value of a diagnostic test is increased if disease screening is based on the presence of clinical signs. Many tests are likely used only in the presence of clinical signs due to their low predictive values or restricted finances.

Vaccination protocols reported from several shelters are not consistent with The Association of Shelter Veterinarians Guidelines for Standards of Care in Animal Shelters which state that vaccines must be used as part of a preventive healthcare plan for shelters in order to prevent deadly disease outbreaks. The Association of Shelter Veterinarians states that pregnancy and mild illness are not contraindications to the administration of core vaccines, though many respondents claimed these as reasons for postponing or foregoing vaccination. Furthermore, modified live vaccines are preferred over killed vaccines even in pregnant animals due to the rapid immune response mounted post-vaccination [13]. Core canine vaccines for intake include canine distemper vaccine, canine parvovirus type 2, canine adenovirus type 2, and the *Bordetella bronchiseptica* vaccine. The rabies vaccine is a core vaccine recommended for all dogs before release from shelter or on intake in long term housing facilities [14]. The American Association of Feline Practitioners states that vaccines for feline panleukopenia virus, feline herpesvirus-1, and feline calicivirus should be considered for all cats immediately upon intake to ensure rapid protection and the rabies vaccine should be used in rabies-endemic areas or where legally mandated or endemic [15].

The results of our study suggest that in Colorado shelters, 53.1% (17/32) of shelters vaccinate dogs and 24.1% (7/29) of shelters vaccinate cats for rabies, with 2 of the 21 vaccinating shelters (9.5%) indicating that they do so upon adoption. A study by Steneroden et al. examining shelter protocols in six states in the Western U.S. suggested a rabies vaccination rate of 45% [10]. Regulations surrounding rabies vaccination in dogs and cats within the state of Colorado vary by county; furthermore, an increase in terrestrial rabies has been reported in northern Colorado in recent years [16] which may explain altered rabies vaccination protocols in this region as a preventive measure. Under the Colorado Practice Act, rabies vaccines are required to be given by a licensed veterinarian. The relative lack of personnel in shelters (47.8% (22/46) of shelters possess veterinarian(s) on staff or contract) may, however, contribute to low rabies vaccination rates in shelters. However, comparing shelters with a veterinarian to those without did not yield a statistically significant difference (*p* = 0.714). Our results suggest that shelters are making vaccination decisions based on an individual animal basis rather than considering population health as the driving force for vaccination upon admittance.

To the authors’ knowledge, this is the first study examining shelter intake procedures at animal shelters in the state of Colorado. However, the study design does possess limitations. A response rate of 29.7% (69/232) was obtained for this study and as such, results may not be representative of the entire Colorado shelter system and this may have adversely affected power in our Fisher’s exact test comparisons of shelter groups. Furthermore, due to the anonymous nature of response, it was not possible to differentiate respondents from non-respondents in relation to shelter demographics. When the survey was disseminated, it was requested that shelter personnel respond and therefore, managers, supervisors, or employees may have responded. Respondents did not export intake and procedure data and thus, some responses may have relied on memory or staff perception. The length of the survey may have contributed to the relatively low response rate. The survey was designed for ease of use so that respondents could skip questions or provide additional information to certain questions. By allowing questions to be skipped, response rates varied by question.

As this study was focused on intake procedures, these data may have underrepresented total vaccine and treatment intakes, as some procedures may have been performed upon adoption, and the number of animals seen per shelter per year did not differentiate between community intakes and transfers. Similarly, our study did not discern at what point during the animal’s stay at the shelter procedures were performed (e.g., intake vs. adoption), unless the respondent voluntarily provided this information. For the portion of the questionnaire focused on euthanasia data, it is important to note that animals considered untreatable, aggressive, or otherwise “unadoptable” and were thus euthanized were likely not included in adoption data, in turn potentially overrepresenting the adoption rate and underrepresenting the euthanasia rate. In questions on disease screening, some respondents voluntarily provided information on non-infectious conditions in the comment box, which may have been due to misunderstanding or the lack of description in the survey on the spectrum of diseases covered. Finally, for questions related to feline disease screening for giardia, roundworm, tapeworm, coccidiosis, and cryptosporidiosis, response rates were low due to survey software malfunction disrupting survey flow.

## 5. Conclusions

The findings of this study provide insight on current/common intake procedures in Colorado animal shelters. The results of this study suggest that half of responding shelters screen for at least one infectious disease, but many shelters more often use diagnostic tests based on the presence of clinical signs rather than routinely screening all incoming animals for particular diseases. Animal origin seems to play an influence in which diseases are screened for upon intake, particularly for dogs. Additionally, vaccination protocols at many shelters are not consistent with The Association of Shelter Veterinarians Guidelines for Standards of Care in Animal Shelters. Comparing information on intake procedures between shelters taking only in-state transfers and those taking in animals from out of state in conjunction with perceived risk about certain diseases highlights the importance of education and awareness surrounding disease incidence and transmission of various infectious agents based on varying animal origin. It may also help guide standard operating procedures for animal shelters and help shape protocols related to disease screening and vaccine administration based on the animal’s history. With increased out-of-state intakes due to ease of travel and increased shelter networking in addition to shifts in vector distribution due to climate change, an improved understanding of which new animal intakes may be at higher risk of infection for various pathogens not only protects other shelter animals, but also shelter staff and potential adopters. In the future, this survey tool may be used on a larger scale in the future to gather knowledge on intake procedures across shelters nationwide.

## Figures and Tables

**Table 1:** Proportion of responding shelters that screen for various infectious diseases in dogs and cats, categorized on the basis of whether or not they accept intakes from out-of-state.

	*n*	Disease Screening	Total	Colorado Only Intakes	Nationwide Intakes	*p*-Value
Canine	44	Any disease screening	50% (22/44)	33.3% (7/21)	65% (15/23)	0.069
	43	Heartworm	37.2% (16/43)	10% (2/20)	69% (14/23)	0.0012
	42	Ehrlichia	4.8% (2/42)	0% (0/21)	9.5% (2/21)	0.488
	43	Lyme	7% (3/43)	0% (0/21)	13.6% (3/22)	0.233
	42	Anaplasma	0% (0/42)	0% (0/21)	0% (0/21)	1
	43	Giardia	18.6% (8/43)	4.8% (1/21)	31.8% (7/22)	0.046
	43	Hookworm	16.3% (7/43)	0% (0/21)	31.8% (7/22)	0.046
	43	Roundworm	18.6% (8/43)	0% (0/21)	36.4% (8/22)	0.004
	43	Whipworm	16.3% (7/43)	0% (0/21)	31.8% (7/22)	0.009
	43	Tapeworm	18.6% (8/43)	4.8% (1/21)	31.8% (7/22)	0.046
	43	Coccidia	9.3% (4/43)	0% (0/21)	18.2% (4/22)	0.108
	43	Flukes	2.3% (1/43)	0% (0/21)	4.5% (1/22)	1
	43	Trichomoniasis	2.3% (1/43)	0% (0/21)	4.5% (1/22)	1
	43	Cryptosporidium	2.3% (1/43)	0% (0/21)	4.5% (1/22)	1
	43	Ringworm	25.6% (11/43)	14.3% (3/21)	36.4% (8/22)	0.162
Feline	41	Any disease screening	43.9% (18/41)	33.3% (6/18)	52.2% (12/23)	0.343
	33	FelV	48.5% (16/33)	33.3% (5/15)	61.1% (11/18)	0.166
	32	FIV	43.8% (14/32)	33.3% (5/15)	52.9% (9/17)	0.308
	33	Heartworm	6.1% (2/33)	0% (0/15)	11.1% (2/18)	0.489
	21	Giardia	4.8% (1/21)	0% (0/11)	10% (1/10)	0.476
	32	Hookworm	9.4% (3/32)	0% (0/15)	17.6% (3/17)	0.229
	22	Roundworm	13.6% (3/22)	0% (0/11)	27.3% (3/11)	0.489
	28	Whipworm	10.7% (3/28)	0% (0/13)	20% (3/15)	0.226
	21	Tapeworm	14.3% (3/21)	0% (0/11)	33.3% (3/10)	0.090
	29	Trichomoniasis	6.9% (2/29)	0% (0/14)	13.3% (2/15)	0.483
	20	Coccidia	10% (2/20)	0% (0/11)	22.2% (2/9)	0.190
	28	Flukes	0% (0/28)	0% (0/14)	0% (0/14)	1
	19	Cryptosporidium	0% (0/19)	0% (0/11)	0% (0/8)	1
	30	Ringworm	40% (12/30)	26.7% (4/15)	53% (8/15)	0.264

**Table 2:** Proportion of respondents indicating which screening method(s) are used in their shelter for various canine diseases.

Disease	Screening Methods	Disease	Screening Methods
**Heartworm**	Serologic antigen test (100%, 17/17) Radiography (23.5%, 4/17) Electrocardiography (0%, 0/17)	**Whipworm**	Fecal flotation (100%, 7/7) Visualization of adults during endoscopic evaluation (0%, 0/7)
**Ehrlichia**	Detection of antibodies by IFA (50%, 1/2) Clinicopathologic findings (0%, 0/2) Blood culture (50%, 1/2) PCR (0%, 0/2)	**Tapeworm**	Fecal flotation (75%, 6/8) Identification of proglottids in feces/vomit (50%, 4/8)
**Lyme**	Serologic antibody test (100%, 3/3) Presence of clinical signs (0%, 0/3) Response to treatment (0%, 0/3)	**Coccidia**	Signalment, clinical signs, history, and structure of oocysts present in feces (66.7%, 4/6) Fecal flotation (66.7%, 4/6)
**Anaplasmosis**	N/A	**Flukes**	Fecal sedimentation (100%, 2/2)
**Giardia**	Fecal flotation (37.5%, 3/8) Fecal smears (75%, 6/8) Fecal ELISA (62.5%, 5/8) Direct fluorescent antibody (0%, 0/8) Washes of duodenal lumen/cytological evaluation of duodenal mucosa (0%, 0/8)	**Trichomoniasis**	Direct fecal smear (100%, 1/1) Culturing in media (0%, 0/1) PCR (0%, 0/1)
**Hookworm**	Fecal flotation (85.7%, 6/7) Signalment and clinical signs (25.6%, 2/7)	**Cryptosporidium**	ELISA assay (100%, 1/1) Fluorescent antibody (0%, 0/1) Sucrose flotation (0%, 1/1)
**Roundworm**	Fecal flotation (100%, 8/8)	**Ringworm**	Clinical signs (e.g. alopecia, pruritis, erythema, crusting of skin) (90.9% (10/11)) Fungal culture (63.6%, 7/11) Wood’s lamp (27.3%, 3/11) Direct microscopic evaluation (27.3%, 3/11)

**Table 3:** Proportion of respondents indicating which screening method(s) are used in their shelter for various feline diseases.

Disease	Screening Methods	Disease	Screening Methods
**Heartworm**	Heartworm serology test (100%, 2/2) Thoracic radiography (0%, 0/2) Echocardiography (0%, 0/2)	**Whipworm**	Fecal flotation (100%, 3/3) Visualization of adults during endoscopic evaluation (0%, 0/3)
**FelV**	Antigen serology test (100%, 15/15)	**Tapeworm**	Identification of proglottids in feces/vomit (37.5%, 9/24) Fecal flotation (25%, 6/24)
**FIV**	Antibody serology test (100%, 15/15)	**Coccidia**	Fecal flotation (32%, 8/25) Signalment, clinical signs, history, and structure of oocysts present in feces (28%, 7/25)
**Giardia**	Fecal flotation (32.1%, 9/28) Fecal smear (21.4%, 6/28) Fecal ELISA (17.9%, 5/28) Direct fluorescent antibody (0%, 0/28) Washes of duodenal lumen/cytological evaluation of duodenal mucosa (0%, 0/28)	**Flukes**	N/A
**Hookworm**	Fecal flotation (100%, 4/4) Signalment and clinical signs (25%, 1/4)	**Trichomoniasis**	Direct fecal smear (66.7%, 2/3) PCR (66.7%, 2/3) Culturing in media (33.3%, 1/3)
**Roundworm**	Fecal flotation (37.5%, 9/24)	**Cryptosporidium**	ELISA assay (2.5%, 2/19)
**Ringworm**	Clinical signs (e.g. alopecia, pruritis, erythema, crusting of skin) (55.3% (16/30)) Wood’s lamp (50%, 15/30) Fungal culture (30%, 9/30) Direct microscopic visualization (13.3%, 4/30)		

**Table 4:** Actions taken upon diagnosis and associated treatment protocols.

Disease	Actions Taken	Treatment
**Canine Heartworm**	Treat and adopt out (93.75%, 15/16) Adopt out, return for treatment (6.25%, 1/16)	Melarsomine dihydrochloride (76.92%, 10/13) Doxycycline (76.92%, 10/13) Exercise restriction (69.23%, 9/13) Macrocyclic lactones (46.15%, 6/13) Adjunct therapy (e.g. steroids, NSAIDs) (38.46%, 5/13) Surgical extraction (15.38%, 2/13)
**Feline Heartworm**	Treat and adopt out (93.75%, 15/16) Adopt out, return for treatment (6.25%, 1/16)	Melarsomine dihydrochloride (76.92%, 10/13) Doxycycline (76.92%, 10/13) Exercise restriction (69.23%, 9/13) Macrocyclic lactones (46.15%, 6/13) Adjunct therapy (e.g. steroids, NSAIDs) (38.46%, 5/13) Surgical extraction (15.38%, 2/13)
**Canine Endoparasites**	Treat and adopt out (90%, 18/20) Euthanize (5%, 1/20) Do not treat and adopt out with medical waiver (0%, 0/20) Do not treat and transfer (0%, 0/20)	Pyrantel (75%, 12/16) Metronidazole (56.3%, 9/16) Fenbendazole (43.8%, 7/16) Praziquantel (37.5%, 6/16) Sulfadimethoxine (18.8%, 3/16) Ponazuril (12.5%, 2/16) Tylosin (12.5%, 2/16) Trimethoprim/sulfonamide (6.3%, 1/16) Moxidectin (6.3%, 1/16) Ronidazole (6.3%, 1/16)
**Feline Endoparasites**	Treat and adopt out (60.6%, 20/33) Do not treat and transfer (6.1%, 2/33) Euthanize (3%, 1/33) Do not treat and adopt out with medical waiver (0%, 0/33)	Pyrantel (54.6%, 12/22) Metronidazole (45.5%, 10/22) Praziquantel (27.3%, 6/22) Fenbendazole (22.7%, 5/22) Sulfadimethoxine (13.6%, 3/22) Ponazuril (13.6%, 3/22) Ronidazole (13.6%, 3/22) Trimethoprim/sulfonamide (9.1%, 2/22) Moxidectin (9.1%, 2/22) Piperazine (4.6%, 1/22) Sulfadimethoxine/ormetoprim (4.6%, 1/22) Selamectin (4.6%, 1/22)

**Table 5:** Respondents indicating “yes” or “uncertain” to a perceived risk were included as positive risk perception.

n	Perceived Risk	CO Only Intakes	Nationwide Intakes	p-Value
39	HW risk in dogs	64.7% (11/17)	86.4% (19/22)	0.142
38	HW risk in cats	58.8% (10/17)	61.9% (13/21)	1
37	Endoparasitic: dogs	94.1% (16/17)	90% (18/20)	1
36	Endoparasitic: cats	87.5% (14/16)	90% (18/20)	1

## Supplementary Materials

The supplementary questionnaire is available online at http://www.mdpi.com/2076-2615/7/5/38/s1.
