# Supplementary material for: Intake Procedures in Colorado Animal Shelters
Source: Animals (Basel). 2017 May 5;7(5):38. doi: 10.3390/ani7050038 (PMC5447920; doi:10.3390/ani7050038)
Supplement: Supplementary file 1 [file animals-07-00038-s001.pdf]

Intake Procedures in Colorado Animal Shelters

The Center for Companion Animal Studies at Colorado State University is looking at intake procedures utilized in animal shelters in Colorado with the aim of understanding what intake procedures are normally used for dogs and cats and under what circumstances intake procedures are altered. Please take a moment to answer these questions. The survey should not last more than 20 minutes. This survey is confidential and each shelter will be assigned a numerical value so responses will not be associated with any particular shelter.

If you have any questions, concerns or would like the survey in a printed form, please contact Rebecca Ruch-Gallie, DVM, MS at 970.297.5000 or rgallie@colostate.edu.

Intake Procedures in Colorado Animal Shelters

General Information

Tell us a little about your shelter. Remember all answers will be held confidentially and not linked to a specific shelter name.

1. Is the shelter you are employed by considered an open or closed admission shelter?

- ○ Open (all dogs & cats are admitted)
- ○ Closed (dogs & cats are admitted on a case-by-case basis)
- ○ Not sure

Other (please specify)

[ ]

2. From which locations do you accept dogs and cats? Mark all that apply.

- ☐ Local community (municipality or county of shelter location)
- ☐ Surrounding communities
- ☐ Within all of Colorado
- ☐ Western States
- ☐ Across the United States
- ☐ Outside the United States
- ☐ Not sure

Other (please specify)

____

3. If you accept dogs and cats from outside communities, which counties within Colorado does the shelter you are employed at accept animals from?

- ☐ ALL
- ☐ Douglas County
- ☐ Broomfield
- ☐ Weld County
- ☐ Garfield County
- ☐ Mesa County
- ☐ Eagle County
- ☐ San Juan County
- ☐ Montrose County
- ☐ Archuleta County
- ☐ Custer County
- ☐ Adams County
- ☐ El Paso County
- ☐ Routt County
- ☐ Grand County
- ☐ Larimer County
- ☐ Summit County
- ☐ Ouray County
- ☐ Arapahoe County

- [ ] La Plata County
- [ ] Elbert County
- [ ] Pitkin County
- [ ] Gilpin County
- [ ] Teller County
- [ ] Pueblo County
- [ ] Dolores County
- [ ] San Miguel County
- [ ] Park County
- [ ] Rio Blanco County
- [ ] Delta County
- [ ] Logan County
- [ ] Gunnison County
- [ ] Chaffee County
- [ ] Bent County
- [ ] Denver County
- [ ] Montezuma County
- [ ] Hinsdale County
- [ ] Crowley County
- [ ] Moffat County
- [ ] Morgan County
- [ ] Kit Carson County
- [ ] Saguache County
- [ ] Alamosa County
- [ ] Yuma County
- [ ] Las Animas County
- [ ] Fremont County
- [ ] Jefferson County
- [ ] Boulder County
- [ ] Phillips County
- [ ] Conejos County

- [ ] Washington County
- [ ] Clear Creek County
- [ ] Rio Grande County
- [ ] Costilla County
- [ ] Lake County
- [ ] Otero County
- [ ] Lincoln County
- [ ] Jackson County
- [ ] Prowers County
- [ ] Sedgwick County
- [ ] Kiowa County
- [ ] Mineral County
- [ ] Huerfano County
- [ ] Baca County
- [ ] Cheyenne County

Intake Procedures in Colorado Animal Shelters

Shelter size and animal management

4. How many dogs and cats are seen at your shelter? Please respond to only one time frame.

Daily

Weekly

Monthly

Quarterly

Annually

5. What percentage of your healthy treatable animals are adopted or re-homed?

6. What percentage of your animals are euthanized (excluding owner-requested euthanasia)?

7. If animals are euthanized at your shelter, please rank the following reasons for euthanasia (with 1 being most frequent).

- Aggression
- Behavioral problems other than aggression (e.g. inappropriate urination, barking, etc.)
- Disease with poor prognosis
- Disease with decent prognosis but expensive treatment
- Limited space and resources
- Other

8. Please list any other reasons your shelter may euthanize that are not included above.

Intake Procedures in Colorado Animal Shelters

Veterinary Care

9. How many, if any, veterinarians does your shelter have on staff or contract?

- None
- 1 part-time
- 2 or more part-time
- 1 full-time
- 1 full-time plus 1 or more part-time
- 2 or more full-time
- 2 or more full-time plus 1 or more part-time

Other (please specify)

10. How many, if any, veterinary technicians does your shelter have on staff or contract?

- ◯ None
- ◯ 1 part-time
- ◯ 2 or more part-time
- ◯ 1 full-time
- ◯ 1 full-time plus 1 or more part-time
- ◯ 2 or more full-time
- ◯ 2 or more full-time plus 1 or more part-time

Other (please specify)

[ ]

11. How many animals are examined by a veterinarian (staff, contract or volunteer) in your shelter upon intake? Please respond to only one time frame.

Daily [ ]

Weekly [ ]

Monthly [ ]

Quarterly [ ]

Annually [ ]

12. How many animals are examined by a veterinary technician (staff, contract or volunteer) in your shelter upon intake? Please choose only one time frame.

Daily [ ]

Weekly [ ]

Monthly [ ]

Quarterly [ ]

Annually [ ]

Intake Procedures in Colorado Animal Shelters

Initial intake procedures for dogs and cats

Please answer the following questions about the initial intake procedures for dogs and cats entering your

shelter.

13. What procedures are performed on ALL dogs and cats upon intake? Please select all that apply.

- [ ] Unique identifier (name and/or number) and record established
- [ ] Physical exam/health status evaluation by veterinarian with documentation in record
- [ ] Physical examination/health evaluation by non-veterinarian with documentation in record
- [ ] Medical history obtained from owner at time of surrender (if possible)
- [ ] Attempts made to locate an animal's owner, including careful screening for identification of microchips
- [ ] Separation of animals by species & age
- [ ] Vaccinations
- [ ] Deworm immediately
- [ ] Other (please specify)

14. What procedures are performed on ONLY SELECT dogs and cats upon intake? Please select all that apply.

- [ ] Unique identifier (name and/or number) and record established
- [ ] Physical exam/health status evaluation by veterinarian with documentation in record
- [ ] Physical examination/health evaluation by non-veterinarian with documentation in record
- [ ] Medical history obtained from owner at time of surrender (if possible)
- [ ] Attempts made to locate an animal's owner, including careful screening for identification of microchips
- [ ] Separation of animals by species & age
- [ ] Vaccinations
- [ ] Deworm immediately
- [ ] Other (please specify)

15. If procedures are not performed on ALL dogs and cats entering the shelter, which of the following impacts whether or not the procedure will be performed? Please select all that apply.

- [ ] Species (dog or cat)
- [ ] Age of animal
- [ ] Sex of animal
- [ ] Reproductive status (neuter or intact) of animal
- [ ] Behavioral assessment
- [ ] Medical assessment
- [ ] Source (owner relinquished, transfer, etc.)
- [ ] Animal's origin (local community, state, etc.)

Other (please specify)

16. If your shelter provides a "Unique identifier (name and/or number) and record established" upon intake, please indicate which items are included in the animal's record.

- [ ] The animal identifier (e.g. name, identification number, etc.)
- [ ] Species
- [ ] Sex
- [ ] Reproductive status (spayed or neutered)
- [ ] Physical description (breed & color)
- [ ] Available medical & behavioral information
- [ ] Results of microchip scan (done multiple times with universal microchip scanner)
- [ ] Microchip number if present
- [ ] Source of animal (e.g. owner surrender, stray, etc.)
- [ ] Dates of entry and departure
- [ ] Outcome (e.g. adoption, transfer, euthanasia, etc.)
- [ ] Other (please specify)

17. If you answered that your shelter vaccinates upon intake, which vaccinations do you provide for dogs? Multiple answers may be selected.

- ☐ Rabies
- ☐ Distemper (alone or in combination)
- ☐ Parvovirus (alone or in combination)
- ☐ Adenovirus-1/hepatitis (alone or in combination)
- ☐ Adenovirus 2 (alone or in combination)
- ☐ Bordetella bronchiseptica injectable (alone or in combination)
- ☐ Bordetella bronchiseptica intranasal or oral (alone or in combination)
- ☐ Lyme disease
- ☐ Leptospirosis
- ☐ Parainfluenza (alone or in combination)
- ☐ Influenza

Other (please specify)

18. If you answered that your shelter vaccinates upon intake, which vaccinations do you provide for cats? Multiple answers may be selected.

- ☐ Rabies
- ☐ Herpevirus-1/Feline viral rhinotracheitis injectable(alone or in combination)
- ☐ Calicivirus injectable(alone or in combination)
- ☐ Panleukopenia injectable(alone or in combination)
- ☐ Herpevirus-1/Feline viral rhinotracheitis intranasal (alone or in combination)
- ☐ Calicivirus intranasal (alone or in combination)
- ☐ Panleukopenia intranasal (alone or in combination)
- ☐ Leukemia virus/FeLV
- ☐ Immunodeficiency virus/FIV
- ☐ Chlamydia
- ☐ Feline Infectious Peritonitis/FIP
- ☐ Ringworm
- ☐ Other (please specify)

Intake Procedures in Colorado Animal Shelters

Canine screening upon intake

19. This questions helps guide whether or not your shelter screens incoming dogs for any of the following diseases:

Heartworm disease
Erlichiosis
Lyme disease
Anaplasmosis
Giardia
Hookworm
Roundworm
Whipworm
Tapeworm
Coccidia
Trematodes/flukes
Trichonella
Cryptosporidium
Ringworm

- ◯ My shelter screens for at least one of the above diseases in dogs upon intake.
- ◯ My shelter does not screen for any the above diseases in dogs upon intake.

Intake Procedures in Colorado Animal Shelters

Screening for heartworm disease in dogs

20. Does the shelter you are employed at screen for heartworm disease in dogs? Multiple answers may be selected.

- ◯ Yes.
- ◯ No,

Intake Procedures in Colorado Animal Shelters

Screening for heartworm disease in dogs

21. What methods are used to screen for heartworm disease?

- [ ] Serologic antigen test.
- [ ] Radiography.
- [ ] Electrocardiography.

Other (please specify)

Intake Procedures in Colorado Animal Shelters

Screening for heartworm disease in dogs

22. Upon learning a dog is positive for heartworm disease, what actions are taken by the shelter?

- ○ Treat and adopt out
- ○ Do not treat & adopt out with medical waiver
- ○ Do not treat and transfer
- ○ Euthanize
- ○ Other (please specify)

Intake Procedures in Colorado Animal Shelters

Treatment of heartworm disease in dogs

23. Upon learning a dog is positive for heartworm disease, what methods of treatment are used?

- ☐ Melarsomine Dihydrochloride
- ☐ Macrocyclic lactones
- ☐ Adjunct therapy (e.g. steroids, NSAIDs)
- ☐ Doxycycline
- ☐ Exercise restriction
- ☐ Surgical extraction

Other (please specify)

Intake Procedures in Colorado Animal Shelters

Screening for erlichiosis in dogs

24. Does the shelter you are employed at screen for erlichiosis in dogs?

- ○ Yes
- ○ No

Intake Procedures in Colorado Animal Shelters

Screening for erlichiosis in dogs

25. What methods are used to screen for erlichiosis?

- ☐ Detection of serum antibodies by use of indirect fluorescence antibody.
- ☐ Clinicopathologic findings such as thrombocytopenia and mild nonregenerative anemia.
- ☐ Blood culture.
- ☐ PCR.

Other (please specify)

Intake Procedures in Colorado Animal Shelters

Screening for Lyme disease in dogs

26. Does the shelter you are employed at screen for Lyme disease in dogs?

- ◯ Yes
- ◯ No

Intake Procedures in Colorado Animal Shelters

Screening for Lyme disease in dogs

27. What methods are used to screen for Lyme disease in dogs?

- ☐ Serologic antibody test
- ☐ Presence of clinical signs
- ☐ Response to treatment

Other (please specify)

Intake Procedures in Colorado Animal Shelters

Screening for anaplasmosis in dogs

28. Does the shelter you are employed at screen for anaplasmosis in dogs?

- ◯ Yes
- ◯ No

Intake Procedures in Colorado Animal Shelters

Screening for anaplasmosis in dogs

29. What methods are used to screen for anaplasmosis in dogs?

- [ ] Serologic antibody test

Other (please specify)

____

Intake Procedures in Colorado Animal Shelters

Screening for giardia in dogs

30. Does the shelter you are employed at screen for giardia in dogs?

- ( ) Yes
- ( ) No

Intake Procedures in Colorado Animal Shelters

Screening for giardia in dogs

31. What methods are used to screen for giardia in dogs? Multiple answers may be selected.

- [ ] Fecal flotation
- [ ] Fecal smears
- [ ] Fecal ELISA (e.g. SNAP Giardia test, IDEXX Laboratories)
- [ ] Direct fluorescent antibody assay
- [ ] Washes of duodenal lumen/cytologic evaluation of duodenal mucosa

Other (please specify)

____

Intake Procedures in Colorado Animal Shelters

Screening for hookworm in dogs

32. Does the shelter you are employed at screen for hookworm in dogs?

- ◯ Yes
- ◯ No

Intake Procedures in Colorado Animal Shelters

Screening for hookworm in dogs

33. What methods are used to screen for hookworm in dogs? Multiple answers may be selected.

- ☐ Fecal flotation
- ☐ Signalment & clinical signs (e.g. iron deficiency anemia in neonates)

Other (please specify)

Intake Procedures in Colorado Animal Shelters

Screening for roundworm in dogs

34. Does the shelter you are employed at screen for roundworm in dogs?.

- ◯ Yes
- ◯ No

Intake Procedures in Colorado Animal Shelters

Screening for roundworm in dogs

35. What methods are used to screen for roundworm in dogs? Multiple answers may be selected.

- ☐ Fecal flotation

Other (please specify)

Intake Procedures in Colorado Animal Shelters

Screening for whipworm in dogs

36. Does the shelter you are employed at screen for whipworm in dogs?

- ◯ Yes
- ◯ No

Intake Procedures in Colorado Animal Shelters

Screening for whipworm in dogs

37. What methods are used to screen for whipworm in dogs? Multiple answers may be selected.

- ☐ Fecal flotation
- ☐ Visualization of adults during endoscopic evaluation

Other (please specify)

Intake Procedures in Colorado Animal Shelters

Screening for tapeworm in dogs

38. Does the shelter you are employed at screen for tapeworm in dogs?

- ◯ Yes
- ◯ No

Intake Procedures in Colorado Animal Shelters

Screening for tapeworm in dogs

39. What methods are used to screen for tapeworm in dogs? Multiple answers may be selected.

- [ ] Fecal flotation
- [ ] Identification of proglottids in feces/vomit

Other (please specify)

Intake Procedures in Colorado Animal Shelters

Screening for coccidiosis in dogs

40. Does the shelter you are employed at screen for the presence of coccidia in dogs?

- ( ) Yes
- ( ) No

Intake Procedures in Colorado Animal Shelters

Screening for coccidiosis in dogs

41. What methods are used to screen for coccidiosis in dogs? Multiple answers may be selected.

- [ ] Signalment, clinical signs, history, and structure of oocysts present in feces
- [ ] Fecal flotation

Other (please specify)

Intake Procedures in Colorado Animal Shelters

Screening for trematodes/flukes in dogs

42. Does the shelter you are employed at screen for the presence of trematodes/flukes in dogs?

- ( ) Yes
- ( ) No

Intake Procedures in Colorado Animal Shelters

Screening for trematodes/flukes in dogs

43. What methods are used to screen for trematodes/flukes in dogs? Multiple answers may be selected.

- [ ] Fecal sedimentation

Other (please specify)

____

Intake Procedures in Colorado Animal Shelters

Screening for trichomoniasis in dogs

44. Does the shelter you are employed at screen for trichomoniasis in dogs?

- ○ Yes
- ○ No

Intake Procedures in Colorado Animal Shelters

Screening for trichomoniasis in dogs

45. What methods are used to screen for trichomoniasis in dogs? Multiple answers may be selected.

- [ ] Direct fecal smear
- [ ] Culturing in media
- [ ] PCR

Other (please specify)

____

Intake Procedures in Colorado Animal Shelters

Screening for cryptosporidiosis in dogs

46. Does the shelter you are employed at screen for cryptosporidiosis in dogs?

- ◯ Yes
- ◯ No

Intake Procedures in Colorado Animal Shelters

Screening for cryptosporidiosis in dogs

47. What methods are used to screen for cryptosporidiosis in dogs? Multiple answers may be selected.

- ☐ ELISA assay
- ☐ Fluorescent antibodies
- ☐ Sucrose flotation

Other (please specify)

Intake Procedures in Colorado Animal Shelters

Management of endoparasitic disease in dogs

48. Upon learning a dog is positive for the endoparasites listed above, what action is taken by the shelter?

- ◯ Treat and adopt out
- ◯ Do not treat & adopt out with medical waiver
- ◯ Do not treat and transfer
- ◯ Euthanize
- ◯ Other (please specify)

Treatment of endoparasitic disease in dogs

49. Which treatment regimen(s) are used by the shelter you are employed at upon finding that a dog is positive for any endoparasitic disease?

- [ ] Fenbendazole
- [ ] Milbemycin oxime
- [ ] Moxidectin
- [ ] Pyrantel
- [ ] Piperazine
- [ ] Sulfadimethoxine
- [ ] Sulfaguanidine
- [ ] Furazolidone
- [ ] Trimethoprim/Sulfonamide
- [ ] Sulfadimethoxine/Ormetoprim
- [ ] Quinacrine
- [ ] Amprolium
- [ ] Amprolium/Sulfadimethoxine
- [ ] Toltrazuril
- [ ] Diclazuril
- [ ] Ponazuril
- [ ] Paromomycin
- [ ] Tylosin
- [ ] Azithromycin
- [ ] Nitazoxanide/Alinia
- [ ] Praziquantel
- [ ] Epsiprantel
- [ ] Metronidazole
- [ ] Albendazole
- [ ] Ronidazole
- [ ] Other (please specify)

Screening for ringworm in dogs

50. Does the shelter you are employed at screen for ringworm in dogs?

- ◯ Yes
- ◯ No

Intake Procedures in Colorado Animal Shelters

Screening for ringworm in dogs

51. What methods are used to screen for ringworm in dogs? Multiple answers may be selected.

- ☐ Wood's lamp
- ☐ Direct microscopic visualization
- ☐ Fungal culture
- ☐ Clinical signs (e.g. alopecia, pruritis, erythema, crusting of skin)

Other (please specify)

52. Does the shelter you are employed at screen for any other diseases in dogs not mentioned above? If so, which disease(s) and how are they diagnosed?

Intake Procedures in Colorado Animal Shelters

Feline screening upon intake

53. This questions helps guide whether or not your shelter screens incoming cats for any of the following diseases:

Feline leukemia
FIV
Heartworm
Giardia
Hook
Round
Whip
Tape
Trichomoniasis
Coccidiosis
Trematodes
Flukes
Crypto
Ringworm

- ◯ My shelter screens for at least one of the above diseases in cats upon intake.
- ◯ My shelter does not screen for any the above diseases in cats upon intake.

Intake Procedures in Colorado Animal Shelters

Screening for feline leukemia in cats

54. Does the shelter you are employed at screen for feline leukemia in cats?

- ◯ Yes
- ◯ No

Intake Procedures in Colorado Animal Shelters

Screening for feline leukemia in cats

55. What methods are used to screen for feline leukemia in cats? Multiple answers may be selected.

- ☐ Antigen serology test

Other (please specify)

Intake Procedures in Colorado Animal Shelters

Screening for FIV in cats

56. Does the shelter you are employed at screen for FIV in cats?

- ◯ Yes
- ◯ No

Intake Procedures in Colorado Animal Shelters

Screening for FIV in cats

57. What methods are used to screen for FIV in cats? Multiple answers may be selected.

- ☐ Antibody serology test

Other (please specify)

____

Intake Procedures in Colorado Animal Shelters

Screening for heartworm disease in cats

58. Does the shelter you are employed at screen for heartworm disease in cats?

- ◯ Yes
- ◯ No

Intake Procedures in Colorado Animal Shelters

Screening for heartworm disease in cats

59. What methods are used to screen for heartworm disease in cats? Multiple answers may be selected.

- [ ] Heartworm serology test
- [ ] Thoracic radiography
- [ ] Echocardiography

Other (please specify)

Intake Procedures in Colorado Animal Shelters

Management of heartworm disease in cats

60. Upon learning a cat is positive for heartworm disease, what actions are taken by the shelter?

- ○ Treat and adopt out
- ○ Do not treat & adopt out with medical waiver
- ○ Euthanize
- ○ Transfer
- ○ Other (please specify)

Intake Procedures in Colorado Animal Shelters

Treatment of heartworm disease in cats

61. If the shelter you are employed at elects to treat cats found heartworm positive, what methods of treatment are used? Multiple answers may be selected.

- [ ] Surgical extraction
- [ ] Prednisone
- [ ] Supportive therapy (e.g. bronchodilators, oxygen therapy, IV fluids, cardiovascular drugs, antibiotics, and nursing care)

Other (please specify)

Intake Procedures in Colorado Animal Shelters

Screening for giardia in cats

62. Does the shelter you are employed at screen for Giardia in cats?

- ○ Yes
- ○ No

Intake Procedures in Colorado Animal Shelters

Screening for giardia in cats

63. What methods are used to screen for giardia in cats? Multiple answers may be selected.

- ☐ Fecal flotation
- ☐ Fecal smears
- ☐ Fecal ELISA (e.g. SNAP Giardia test, IDEXX Laboratories)
- ☐ Washes of duodenal lumen/cytologic evaluation of duodenal mucosa
- ☐ Direct fluorescent antibody assay
- ☐ Other (please specify)

Intake Procedures in Colorado Animal Shelters

Screening for hookworm in cats

64. Does the shelter you are employed at screen for hookworm in cats?

- ○ Yes
- ○ No

Intake Procedures in Colorado Animal Shelters

Screening for hookworm in cats

65. What methods are used to screen for hookworm in cats? Multiple answers may be selected.

- [ ] Fecal flotation
- [ ] Signalment & clinical signs (e.g. iron deficiency anemia in neonates)
- [ ] Other (please specify)

Intake Procedures in Colorado Animal Shelters

Screening for roundworm in cats

66. Does the shelter you are employed at screen for roundworm in cats?

- ○ Yes
- ○ No

Intake Procedures in Colorado Animal Shelters

Screening for roundworm in cats

67. What methods are used to screen for roundworm in cats? Multiple answers may be selected.

- [ ] Fecal flotation
- [ ] Other (please specify)

Intake Procedures in Colorado Animal Shelters

Screening for whipworm in cats

68. Does the shelter you are employed at screen for whipworm in cats?

- ◯ Yes
- ◯ No

Intake Procedures in Colorado Animal Shelters

Screening for whipworm in cats

69. What methods are used to screen for whipworm in cats? Multiple answers may be selected.

- ☐ Fecal flotation
- ☐ Visualization of adults during endoscopic evaluation
- ☐ Other (please specify)

Intake Procedures in Colorado Animal Shelters

Screening for tapeworm in cats

70. Does the shelter you are employed at screen for tapeworm in cats?

- ◯ Yes
- ◯ No

Intake Procedures in Colorado Animal Shelters

Screening for tapeworm in cats

71. What methods are used to screen for tapeworm in cats? Multiple answers may be selected.

☐ Fecal flotation

☐ Identification of proglottids in feces/vomit

☐ Other (please specify)

Intake Procedures in Colorado Animal Shelters

Screening for trichomoniasis in cats

72. Does the shelter you are employed at screen for trichomoniasis in cats?

○ Yes

○ No

Intake Procedures in Colorado Animal Shelters

Screening for trichomoniasis in cats

73. What methods are used to screen for trichomoniasis in cats? Multiple answers may be selected.

☐ Direct fecal smear

☐ Culturing in media

☐ PCR

☐ Other (please specify)

Intake Procedures in Colorado Animal Shelters

Screening for coccidiosis in cats

74. Does the shelter you are employed at screen for the presence of coccidia in cats?

- ◯ Yes
- ◯ No

Intake Procedures in Colorado Animal Shelters

Screening for coccidiosis in cats

75. What methods are used to screen for coccidiosis in cats? Multiple answers may be selected.

- ☐ Signalment, clinical signs, history, and structure of oocysts present in feces
- ☐ Fecal flotation
- ☐ Other (please specify)

Intake Procedures in Colorado Animal Shelters

Screening for trematodes/flukes in cats

76. Does the shelter you are employed at screen for the presence of trematodes/flukes in cats?

- ◯ Yes
- ◯ No

Intake Procedures in Colorado Animal Shelters

Screening for trematodes/flukes in cats

77. What methods are used to screen for the presence of trematodes/flukes in cats? Multiple answers may be selected.

- ☐ Fecal sedimentatio
- ☐ Other (please specify)

Intake Procedures in Colorado Animal Shelters

Screening for cryptosporidiosis in cats

78. Does the shelter you are employed at screen for cryptosporidiosis in cats?

- ◯ Yes
- ◯ No

Intake Procedures in Colorado Animal Shelters

Screening for cryptosporidiosis in cats

79. What methods are used to screen for cryptosporidiosis in cats? Multiple answers may be selected.

- ☐ ELISA assay
- ☐ Fluorescent antibodies
- ☐ Sucrose flotation
- ☐ Other (please specify)

Intake Procedures in Colorado Animal Shelters

Management of endoparasitic disease in cats

80. Upon learning a dog is positive for the endoparasites listed above, what action is taken by the shelter?

- ◯ Treat and adopt out
- ◯ Do not treat & adopt out with medical waiver
- ◯ Do not treat and transfer
- ◯ Euthanize
- ◯ Other (please specify)

Intake Procedures in Colorado Animal Shelters

Treatment of endoparasitic disease in cats

81. Which treatment regimen(s) are used by the shelter you are employed at upon finding that a cat is positive for any endoparasitic disease?

- [ ] Fenbendazole
- [ ] Milbemycin oxime
- [ ] Moxidectin
- [ ] Pyrantel
- [ ] Selamectin
- [ ] Piperazine
- [ ] Sulfadimethoxine
- [ ] Sulfaguanidine
- [ ] Furazolidone
- [ ] Trimethoprim/Sulfonamide
- [ ] Sulfadimethoxine/Ormetoprim
- [ ] Quinacrine
- [ ] Amprolium
- [ ] Amprolium/Sulfadimethoxine
- [ ] Toltrazuril
- [ ] Diclazuril
- [ ] Ponazuril
- [ ] Praziquantel
- [ ] Epsiprantel
- [ ] Metronidazole
- [ ] Albendazol
- [ ] Ronidazole
- [ ] Other (please specify)

Intake Procedures in Colorado Animal Shelters

Screening for ringworm in cats

82. Does the shelter you are employed at screen for ringworm in cats?

- ○ Yes
- ○ No

Intake Procedures in Colorado Animal Shelters

Screening for ringworm in cats

83. What methods are used to screen for ringworm in cats? Multiple answers may be selected.

- ☐ Wood's lamp
- ☐ Direct microscopic visualization
- ☐ Fungal culture
- ☐ Clinical signs (e.g. alopecia, pruritis, erythema, crusting of skin)
- ☐ Other (please specify)

84. Does the shelter you are employed at screen for any other diseases in cats not mentioned above? If so, which disease(s) and how are they diagnosed?

Intake Procedures in Colorado Animal Shelters

Reasons for withholding vaccination

85. If dogs and cats do not receive core vaccinations upon intake in certain situations, it is for the following reasons. Multiple answers may be selected.

- [ ] The animal is pregnant.
- [ ] The animal has mild existing illness.
- [ ] The animal is too young to vaccinate.
- [ ] The animal is too old to vaccinate.
- [ ] Animals always receive core vaccinations upon intake.
- [ ] Other (please specify)

Intake Procedures in Colorado Animal Shelters

Questions related to heartworm disease

86. If your shelter accepts transfers from any colored section on the map, please select the color(s) of states that you require a heartworm screening test be performed upon intake.

- [ ] Blue
- [ ] Green
- [ ] Yellow
- [ ] The shelter I am employed at does not accept animals from outside the state of Colorado.

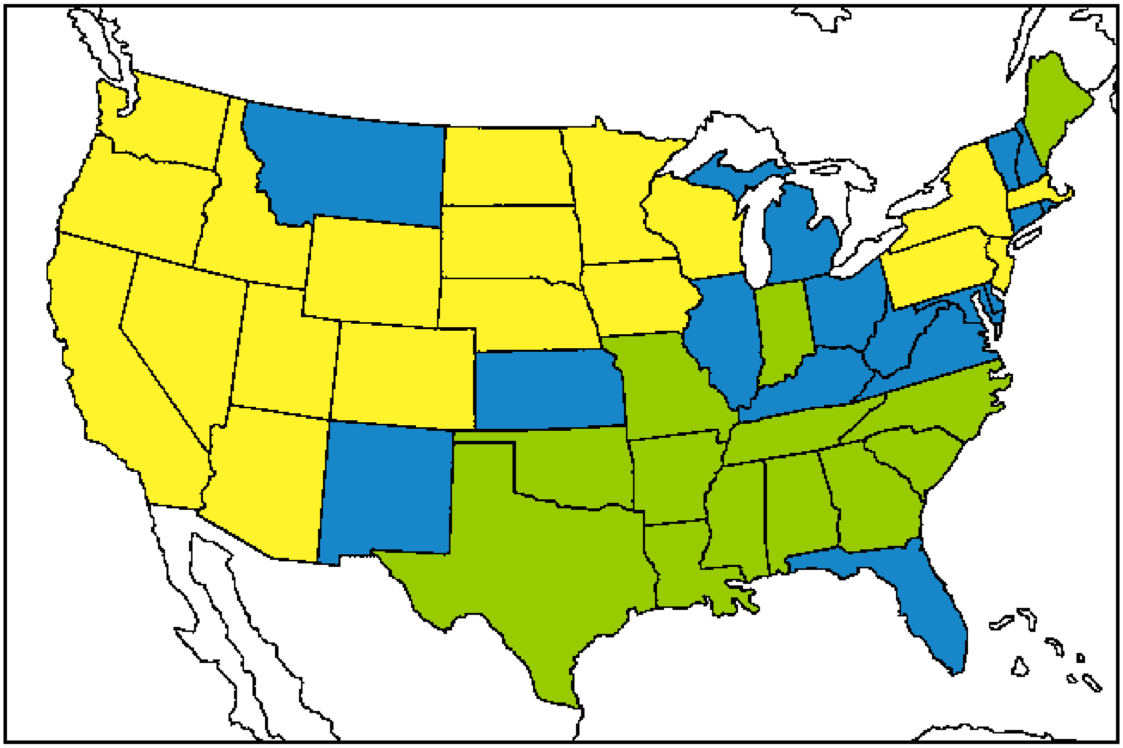

Intake Procedures in Colorado Animal Shelters

Questions related to the diagnosis and management of endoparasitic (worm) disease

87. If your shelter accepts transfers from any of the colored areas, please select the color(s) of states that you require an endoparasitic (worm) screening test be done upon intake. If you do not screen for endoparasites, skip to Question 54.

- [ ] Blue
- [ ] Green
- [ ] Orange
- [ ] The shelter I am employed at does not accept animals from outside the state of Colorado.

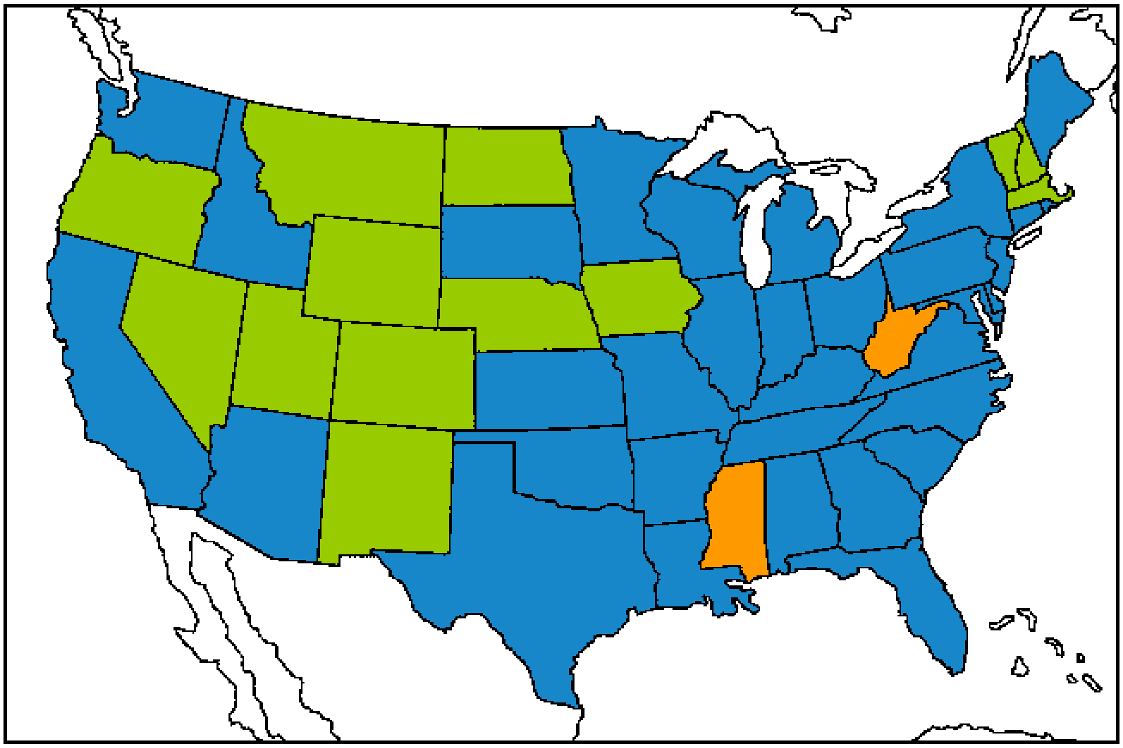

88. Do you perceive heartworm disease as a risk to the canine population in Colorado?

- ☐ Yes
- ☐ No
- ☐ Not sure

89. Do you perceive heartworm disease as a risk to the feline population in Colorado?

- ☐ Yes
- ☐ No
- ☐ Not sure

90. Do you perceive endoparasitic disease as a risk to the canine population in Colorado?

- ◯ Yes
- ◯ No
- ◯ Not sure

91. Do you perceive endoparasitic disease as a risk to the feline population in Colorado?

- ◯ Yes
- ◯ No
- ◯ Not sure
